# Brain MRI Pattern Recognition Translated to Clinical Scenarios

**DOI:** 10.3389/fnins.2017.00578

**Published:** 2017-10-20

**Authors:** Andreia V. Faria, Zifei Liang, Michael I. Miller, Susumu Mori

**Affiliations:** ^1^Department of Radiology, Johns Hopkins University, Baltimore, MD, United States; ^2^Department of Radiology, New York University, New York, NY, United States; ^3^Department of Biomedical Engineering, Johns Hopkins University, Baltimore, MD, United States

**Keywords:** precision medicine, pattern recognition, quantitative MRI, computer aid, automated MRI analysis

## Abstract

We explored the performance of structure-based computational analysis in four neurodegenerative conditions [Ataxia (AT, *n* = 16), Huntington's Disease (HD, *n* = 52), Alzheimer's Disease (AD, *n* = 66), and Primary Progressive Aphasia (PPA, *n* = 50)], all characterized by brain atrophy. The independent variables were the volumes of 283 anatomical areas, derived from automated segmentation of T1-high resolution brain MRIs. The segmentation based volumetric quantification reduces image dimensionality from the voxel level [on the order of O(10^6^)] to anatomical structures [O(10^2^)] for subsequent statistical analysis. We evaluated the effectiveness of this approach on extracting anatomical features, already described by human experience and a priori biological knowledge, in specific scenarios: (1) when pathologies were relatively homogeneous, with evident image alterations (e.g., AT); (2) when the time course was highly correlated with the anatomical changes (e.g., HD), an analogy for prediction; (3) when the pathology embraced heterogeneous phenotypes (e.g., AD) so the classification was less efficient but, in compensation, anatomical and clinical information were less redundant; and (4) when the entity was composed of multiple subgroups that had some degree of anatomical representation (e.g., PPA), showing the potential of this method for the clustering of more homogeneous phenotypes that can be of clinical importance. Using the structure-based quantification and simple linear classifiers (partial least square), we achieve 87.5 and 73% of accuracy on differentiating AT and pre-symptomatic HD patents from controls, respectively. More importantly, the anatomical features automatically revealed by the classifiers agreed with the patterns previously described on these pathologies. The accuracy was lower (68%) on differentiating AD from controls, as AD does not display a clear anatomical phenotype. On the other hand, the method identified PPA clinical phenotypes and their respective anatomical signatures. Although most of the data are presented here as proof of concept in simulated clinical scenarios, structure-based analysis was potentially effective in characterizing phenotypes, retrieving relevant anatomical features, predicting prognosis, and aiding diagnosis, with the advantage of being easily translatable to clinics and understandable biologically.

## Introduction

A longtime dream of clinicians is to use computational tools for aiding decisions. Like using the spelling and grammar checkers when writing a text or Google for searching, clinical computational tools would neither define purposes nor change goals, but add a higher level of quality and speed to the results. There are three must-haves for computational-aid tools: speed, automation, and, of course, efficacy. The development of such tools for medical records and imaging, in particular, is extremely complex, involving knowledge in multiple domains. Consequently, more than two decades after the initial attempts (for review and perspectives see Orphanoudakis et al., 1996; Akgul et al., 2011; Hwang et al., 2012; Kalpathy-Cramer et al., 2015; Pinho et al., 2017; Spanier et al., 2017), no system is yet adequately suited for practical daily use. The key to translating the computational models to radiological practice is to resolve the so-called semantic gap: “the differences between image similarity on the high level of human perception and the low level of a few numbers” (Depeursinge et al., 2011). Three basic steps are involved: precise quantification, optimal feature selection and combination, and, eventually, meaningful applications and testing.

The first step, image quantification, is straightforward if one is simply interested in the intensity of a given voxel. What is not simple, however, is to extract some biological meaning from the noisy voxel-by-voxel information, which can be of the order of 10^6^, considering only T1-weighted images, one of the multiple MRI contrasts. There are numerous papers on voxel-based analysis (VBA) in which human involvement is eliminated on the assumption that a human being's ability to detect abnormality is neither sensitive nor reliable. A PubMed search for “VBA,” “brain,” and “MRI” results in more than 2,300 publications in the last 10 years. These studies provide a wealth of descriptive imaging results that are usually not perceptive at an individual level and fail to be translated to clinical practice, which meanwhile, remains supported by human judgment. If we flip this approach 180° by asking: Can a computational approach describe abnormalities that agree with human perception?, we find the number of publications to be surprisingly small. A PubMed search for “structure-based analysis” or “atlas-based analysis,” “brain,” and “MRI” results in fewer than 200 publications in the last 10 years. An old strategy to replicate human perception is to group voxels in regions of interest (ROIs) and label them according to existing anatomical knowledge. For example, all the voxels associated with certain x, y, z coordinates are called “thalamus,” or “frontal lobe,” or “internal capsule,” and so on. This is what radiologists do, increasing the signal-to-noise ratio and adding a biological domain to their subjective analysis. However, objectively quantifying, structurizing, and recording the information for subsequent use is much more complex. In addition, defining ROIs in multiple subjects multidimensionally is just not feasible; precise automated tools are vital.

This structure-based analysis is linked to the second step to solve the semantic gap: the feature selection and combination. Here, two components are essential: the existing knowledge of normal and abnormal patterns and the ability to recognize these patterns in future patients. For example, when a patient has striatum atrophy and motor disabilities, Huntington's Disease (HD) is a possible diagnosis because physicians learned that these two features are associated with this disease. In addition to centuries of pathophysiological knowledge, what is hidden behind this apparently simple conclusion is an enormous amount of comprehension about normal variation. In order to conclude that those regions, in an individual of a certain age and gender, are smaller than expected, an analysis of multiple granularity levels (looking to the caudate, or the basal ganglia, or the deep gray matter, or the lobe, or the whole brain), and multiple image domains (volume, intensity, shapes), and finally the combination of features in different fields (clinical and imaging) are necessary. This leads to the amazing capability of pattern recognition that humans have and that machine-learning methods try to replicate.

Finally, even if we are able to quantify structures precisely in different levels and domains, to compare individual cases with large and variable normal and pathological databases, and to extract and combine important features efficiently, we still have to suit the computer-aid tools to the appropriate applications and test them. If the goal is a diagnostic-aid tool, this may be the most challenging step because the gold-standard is the clinical diagnosis, which does not necessarily reflect the actual situation. In addition, the correlation between pathology and anatomy may be weak or indirect. This is usually the case in pathologies in which the anatomical changes are subtle or happen later, or when the time course is unknown, or in those that embrace heterogeneous phenotypes. These cases are challenging and may reduce the efficiency of classification models, but they also offer an opportunity to design tools for binning a given entity into subgroups, for example, that may be of clinical relevance.

Previously, our group and others advanced in the first two steps (quantification and feature extraction). The brain quantification and segmentation accuracy improved drastically in this decade due to the advances in multi-atlas technologies (Warfield et al., 2004; Artaechevarria et al., 2009; Langerak et al., 2010; Lotjonen et al., 2010; Sabuncu et al., 2010; van Rikxoort et al., 2010; Jia et al., 2012; Wang et al., 2013), allowing use of state-of-the-art techniques for quantification and extraction of clinically meaningful image features. We confirmed the accuracy of these techniques in different populations and protocols (Liang et al., 2015). We then tested whether the structured anatomical data extracted actually captured the anatomical features that can be perceived by trained clinicians (Faria et al., 2015). In the present study, we advance to the next step and report progress on feature selection, combination, and classification, showing the potential of structure-based analysis for computer-aided decisions.

This study focused on the brain MRIs of patients with these neurodegenerative conditions: Ataxia (AT), Huntington's Disease (HD), Alzheimer's Disease (AD), and Primary Progressive Aphasia (PPA). Briefly, Ataxia, or more specifically, the Spinocerebellar ataxia type 6 (SCA-6) which is considered here, is an autosomal dominant disorder that is characterized by a slowly progressive cerebellar ataxia, dysarthria and nystagmus (Zhuchenko et al., 1997). The cerebellar atrophy, demonstrated by several prior MRI studies, is a constant (Butteriss et al., 2005) and relates with clinical manifestations (Eichler et al., 2011).

HD is a progressive lethal neurodegenerative disorder characterized by movement disorders and progressive cognitive and psychological manifestations (Huntington, 1872). The anatomical hallmark of HD is striatal atrophy. Although the atrophy may start as early as 15 years before the onset of motor symptoms, and continue through the pre-manifest period (Tabrizi et al., 2009, 2012, 2013; Paulsen et al., 2014a,b), it is mostly undetectable by clinical evaluation of MRIs, at individual level, in pre-symptomatic patients. The early quantitative characterization of the atrophy, both at group and individual level, is an important piece of information for the development of disease-modifying treatments (Faria et al., 2016; Wu et al., 2016).

Alzheimer disease (AD) is a chronic neurodegenerative disease characterized by short-term memory loss in the early disease stages and progressive cognitive and functional deficits as the disease advances. It is actually not a single disease but a clinically, anatomically and biologically heterogeneous disorder encompassing a wide spectrum of cognitive and anatomical profiles (Zhang et al., 2016). Although a classical pattern of atrophy is reported for AD as a group, first noticeable in the medial temporal lobe (including hippocampus and entorhinal cortex), eventually spreading through the remainder of the brain (Apostolova et al., 2007), this pattern is not highly discriminant at individual level (Frisoni et al., 2017). In addition, the atrophy is usually clinically evident long after the cognitive deficits start. The heterogeneity of phenotypes and subtleness of early anatomical changes are extra challenges for the development of therapeutics and prognostic models.

Primary progressive aphasia (PPA) is a clinical syndrome characterized by insidious progressive language impairment that is initially unaccompanied by other cognitive deficits (Mesulam, 1982). It is caused by various neurodegenerative diseases and has a highly variable course. There are three main variants that are distinguished by their key features and supporting brain imaging characteristics, which are generally associated with distinct underlying pathologies (Gorno-Tempini et al., 2011): agramatic (Av) is supported by left posterior frontal and (Zhuchenko et al., 1997) insular atrophy; semantic (Sv) is associated with left greater than right anterior and inferior temporal atrophy; logopenic (Lv) is associated with posterior temporal and inferior parietal atrophy (Rohrer and Rosen, 2013; Wilson et al., 2016). The identification of the variant provides some clues regarding the subsequent course (Leyton et al., 2016), and would be of great value for prognosis in the initial stages. However, the early classification is particularly challenging because the clinical deficits are common to all three variants and the anatomical changes are still clinically silent. Methods for phenotypically characterization, particularly at early phases, would be of great assistance.

The choice of these clinical entities was due to the fact that the common feature (atrophy) varies in extension and location, providing an appropriate dynamic range of abnormalities. In addition, the atrophy is mostly visible, which enables validation by qualitative human evaluation. The overall goal of this study was to test the performance of structure-based computational analysis on extracting anatomical features, already described by human experience and a priori biological knowledge, in specific patient populations. The variables in question were the volumes of 283 structures. We showed the potential of the structure-based analysis on characterization and classification (1) when pathologies were relatively homogeneous, with evident image alterations (e.g., Ataxias); (2) when the time course was highly correlated with the anatomical changes (e.g., HD), an analogy for prediction; (3) when the pathology embraced heterogeneous phenotypes (e.g., AD) so the classification was less efficient but, in compensation, anatomical and clinical information were less redundant; and (4) when the entity was composed of multiple subgroups that had some degree of anatomical representation (e.g., Primary Progressive Aphasia), showing the potential of this method for the clustering of more homogeneous phenotypes that can be of clinical importance.

## Materials and methods

### Database

The overall goal was to test the performance of structure-based computational analysis in extracting anatomical features, previously described by human experience and a priori biological knowledge, in specific patient populations.

The data consisted of high-resolution T1-weighted brain MRIs (MPRAGE), for five groups of individuals: healthy individuals (controls, *n* = 208), AT (*n* = 16), HD (*n* = 52), AD (*n* = 66), and PPA (*n* = 50) (Table 1). The data from healthy individuals (controls) were obtained from three sources: (1) internal datasets from Johns Hopkins University (JHU), (2) International Consortium for Brain Mapping (ICBM, loni.usc.edu/ICBM), and (3) the AD Neuroimaging Initiative (ADNI, adni.loni.usc.edu). The control dataset included more than 10 different protocols (including different machine manufacturers, strength of magnetic field, and resolution), thus replicating the heterogeneity encountered in clinical scenarios. Individuals with AT were from JHU and had spinocerebellar ataxia type 6 (SAC6). Individuals with HD, also from JHU, were grouped into three different stages, according to their CAG-Age Product (CAP) scores (Penney et al., 1997) and clinical symptoms: pre-symptomatic far from onset (*n* = 23), pre-symptomatic close to onset (*n* = 16), and early symptomatic (*n* = 13). Individuals with AD, from JHU and ADNI, were diagnosed according to new clinical guidelines (Albert et al., 2011; Jack et al., 2011; McKhann et al., 2011; Sperling et al., 2011). Individuals with PPA, from JHU, were diagnosed and classified into three variants: logopenic (Lv, *n* = 18), semantic (Sv, *n* = 16), and agrammatic (Av, *n* = 16), based on current clinical guidelines (Mesulam, 1982; Gorno-Tempini et al., 2011). All the data had previously been de-identified, and the participants consented to enrolling by written consent.

**Table 1:** Demographic and protocol information.

**Group**	**Sample**	**Age range (Years)**	**Mean age ± St.dev. (Years)**	**Male/Female**	**Protocols (Manufacturer, Field Strength (T), Voxel Size (mm), Sample)**
**Controls**	208	20–95	57.9 ± 18.8	98/110	Phillips, 1.5, 1 × 0.875 × 0.875, 28
					Phillips, 1.5, 1.2 × 0.94 × 0.94, 9
					Phillips, 3, 1 × 1 × 1, 7
					Phillips, 3, 1.2 × 1 × 1, 26
					Phillips, 3, 0.9 × 0.9 × 0.9, 52
					Phillips, 3, 1.1 × 0.83 × 0.83, 16
					GE, 1.5, 1.2 × 0.94 × 0.94, 6
					GE, 3, 1.2 × 1.02 × 1.02, 6
					Siemens, 1.5, 1.2 × 1.25 × 1.25, 7
					Siemens, 3, 1 × 1 × 1, 28
					Siemens, 3, 1.2 × 1 × 1, 23
**Ataxia**	16	48–73	60.8 ± 6.8	13/3	Phillips, 3, 1.1 × 0.83 × 0.83, 16
**HD**					
Far from onset	23	21–51	36.8 ± 9.7	10/13	
Near to onset	16	20–55	45.1 ± 8.6	13/3	Phillips, 3, 0.9 × 0.9 × 0.9, 52
Early symptoms	13	30–59	50.8 ± 7.9	7/6	
**AD**	66	55–93	74 ± 10.5	40/26	Siemens, 3, 1.2 × 1 × 1, 27
					Siemens, 1.5, 1.2 × 1.25 × 1.25, 7
					Phillips, 1.5, 1.2 × 0.94 × 0.94, 7
					Phillips, 3, 1.2 × 1 × 1, 8
					GE, 1.5, 1.2 × 0.94 × 0.94, 9
					GE, 3, 1.2 × 1.02 × 1.02, 8
**PPA**					
Lv	18	51–79	68.3 ± 5.4	10/8	Siemens, 3, 1 × 1 × 1, 21
Sv	16	57–77	65.5 ± 6.5	11/5	Phillips, 3, 1.2 × 1 × 1, 29
Av	16	48–84	68.2 ± 10.7	9/7	

### Image processing

In the present study, quantification of regional brain volume was performed on a structural level, which involved the mapping of each brain to 29 templates in which the structures in question had previously been labeled. The brain mapping was performed with large deformation diffeomorphic metric mapping (LDDMM) (Wang et al., 2007; Ceritoglu et al., 2009; Djamanakova et al., 2013). Inversely, the labels were warped to each subject space and then fused by a likelihood fusion algorithm, which took into account both the location and intensity information of each label (Langerak et al., 2010; Sabuncu et al., 2010; Wang et al., 2013). The details of this method, the atlas creation, and the validation in diverse protocols and anatomical phenotypes are described in our previous publications (Tang et al., 2013; Liang et al., 2015; Ma et al., 2015; Wu et al., 2016).

**Figure 1:**
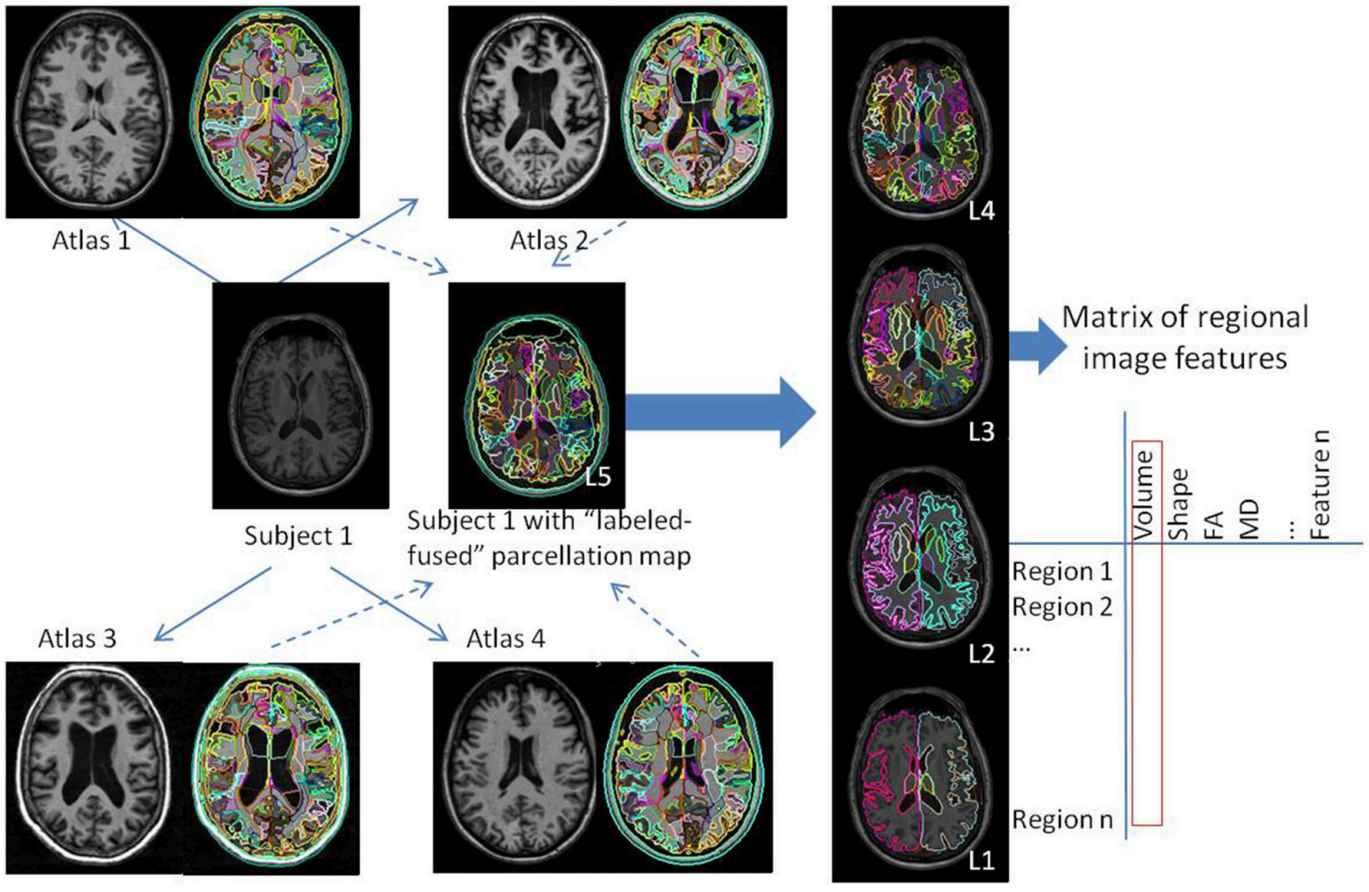
Schematic representation of the automated image parcellation using a multi-atlas likelihood fusion (MALF) algorithm. Each brain image is mapped to each atlas, and the pre-defined labels are correlated with each original brain. The labels can be grouped into five ontological hierarchical levels (L1–L5). By this process, the images are converted to matrices of structures by image features; in the present study, we used the regional volumes.

By this multi-atlas automated brain segmentation tool, the raw images, which consisted of more than 1 million voxels were converted to 286 structural representations, of which the volumes were measured. Based on the hierarchical relationship defined in the atlas, these structures can be combined to create five ontological levels with 8–19–53–125–286 structures respectively (Figure 1). Details of the hierarchical-ontological grouping are found in our previous publications (Djamanakova et al., 2014; Wu et al., 2016). One of the reasons for choosing the structure-based multi-level design is that the physician's analysis does not operate at the voxel level, but at the structural level, migrating freely along the hierarchy. The choice of level is a trade-off between regional specificity and noise: in higher levels, more structures are defined and spatial specificity increases, yet noise also increases. In hypothesis-driven studies, the choice of the level depends on the interest in a given structure. In data-driven studies, the data can be analyzed using all ontological levels combined, or at each level independently. Our present analyses were performed according to the latter approach.

### Statistical analysis and outputs

We used partial least square—discriminant analysis (PLS-DA) to classify individuals in three different analyses: (1) AT vs. controls, (2) HD vs. controls, and (3) AD vs. controls. As many different protocols as possible were included for each analysis, yet keeping the individuals paired by age, gender, and image protocol in each group compared. The PLS-DA inputs were the regional volumes of brain structures in the five ontological levels, normalized by the intracranial volume. As the classification accuracy increased with the level of granularity and converged at level 3, the results are reported at this level. Level 3 is a medium level of granularity, where the whole brain is segmented in lobes, deep gray matter, major deep white matter structures, ventricles, and sulci (Djamanakova et al., 2014). It matches well the radiologists reading (Faria et al., 2015), and the segmentation reproducibility is high (Djamanakova et al., 2013; Faria et al., 2015; Liang et al., 2015).

We opted for using simple linear classifiers to reduce the chance of overfitting, increase the potential for generalization of the results, and facilitate the translation to clinical practice, which is our aim, rather than the greatness of the classification. We could have obtained higher classification accuracy using more elaborate classifiers (such as a support vector machine and black-box models). Briefly, PLS is the least restrictive extension of the multiple linear regression models, therefore applicable to situations where the number of predictor variables exceeds the number of observations. As in the principal component analysis (PCA), the scores, or components, are the sets of values of linearly uncorrelated variables and the regression coefficients (loadings or weights) reflect the importance of the predictor variables in the model.

In each analysis, the samples were divided in training set, in which the classifier was built, and test set, in which the accuracy was tested. The validation in an independent test set reduces the impact of overfitting by biased variable selection and results in more realistic classification accuracy. In addition to the classifier accuracy, the outputs of interest were (1) the anatomical features important for the classification (related to the PLS loading weights) or, in other words, the regional pattern of atrophy that characterizes each group, and (2) the individual's chances of belonging to different groups, which can be of direct importance for clinical guidance. Secondary outputs of interest are (1) the distance among individuals in the principal component space, which can be used for image retrieval of individuals with similar phenotypes, and (2) the individual z-score maps of atrophy.

In the case of PPA, we qualitatively explored a possible natural segregation among the phenotypes with PCA. The inputs were, again, the regional volumes of brain structures, normalized by the intracranial volume. We then assessed the potential of our tools on subdividing groups according to anatomical phenotype, using hierarchical clustering.

## Results

### Ataxia: extraction of homogeneous and noticeable image features

**Figure 2:**
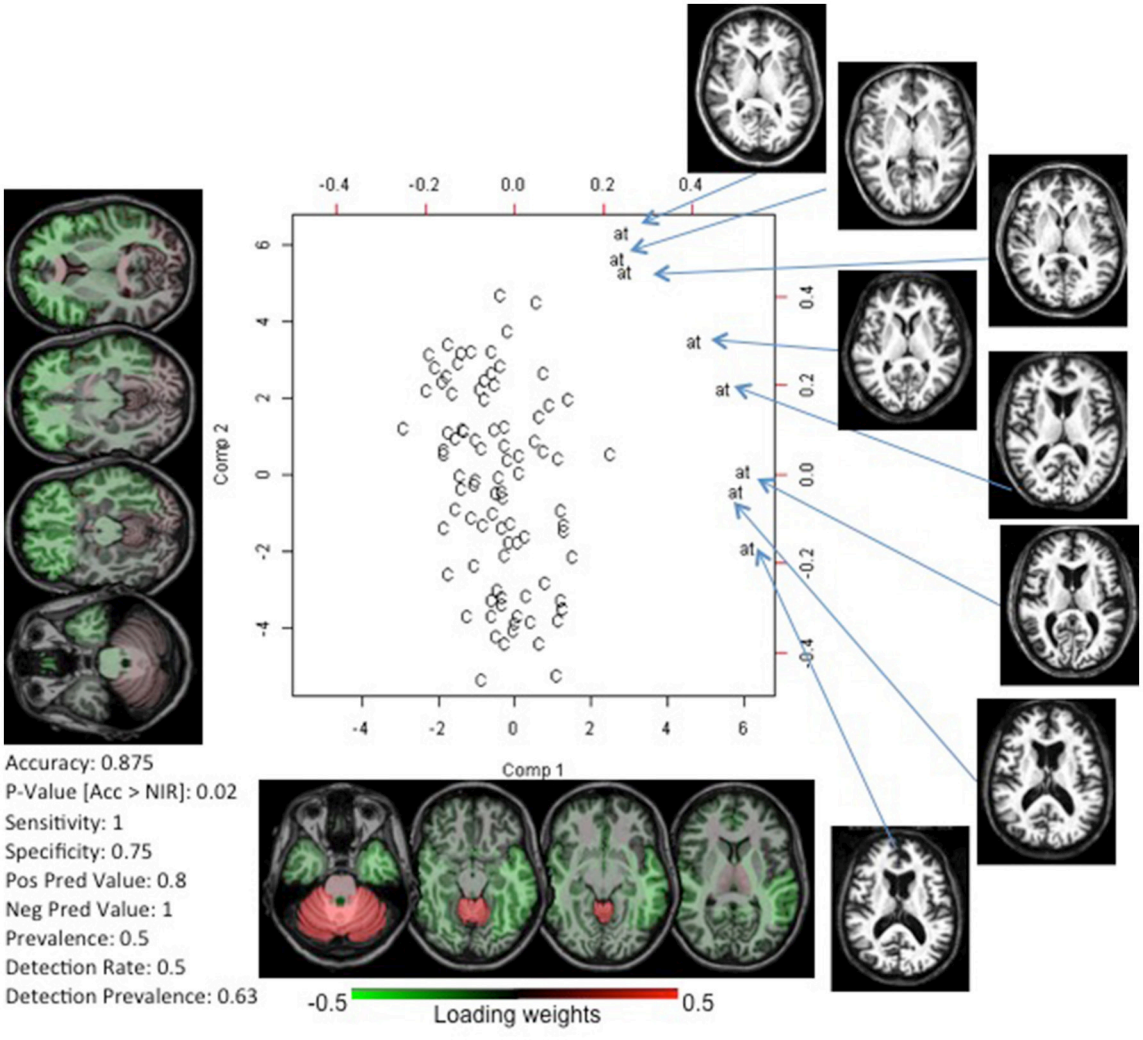
Biplot of scores and loadings from a PLS-DA analysis between controls (C) and patients with ataxia (at). The loading weights of the regional volumes, or the importance of regional atrophy in the classifier, are color-coded on the axial MRIs (radiological view). This gives, at a glance, a snapshot of the important features of the disease. In this case, the volume of the cerebellum (component 1) and brainstem/mesencephalon (component 2) had the highest absolute weights, in agreement with the physiopathology of ataxia. At the bottom left, the classifier accuracy in an external test set is reported. The actual brain images of the patients used in the model are shown.

The analysis performed on 16 individuals with ataxia (8 for training, 8 for testing; Supplementary Table), and controls paired by age, gender, and image protocol achieved accuracy of 0.875 in differentiating individuals with AT from controls. Figure 2 shows the PLS-DA plot (scores vs. loadings) and the two components used by the classifier. Component 1 is mostly responsible for the segregation between the two groups. The cerebellum had the highest loading, i.e., the cerebellar atrophy played a major role on the classification, in agreement with the well-known and apparent cerebellar atrophy in ataxia. The highest absolute loadings of component 2 are diffusely distributed among the frontal, temporal and parietal lobes; it directly correlated with the degree of atrophy on these lobes, as measured by their volumetric z-score (Pearson rho of 0.72, 0.67, 0.61 for frontal, temporal, and parietal, respectively), and inversely correlated with age (rho = −0.77). Therefore, we infer that component 2 reflects age-related atrophy in individuals with AT.

### Huntington's disease: prediction

**Figure 3:**
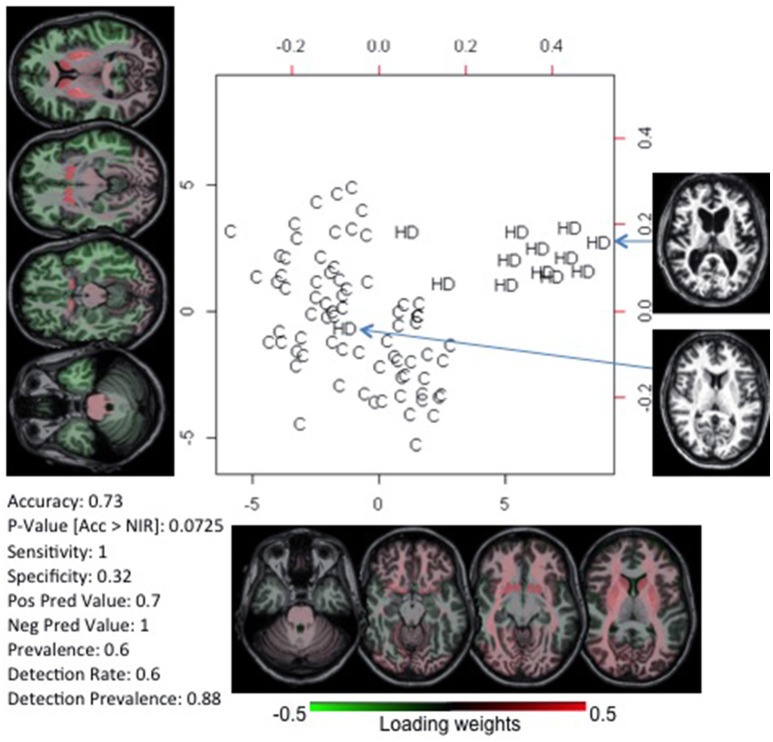
PLS-DA biplot of controls (C) and patients with early symptoms of Huntington's Disease (HD). The deep gray matter has the highest absolute weight, in agreement with the anatomical pattern typically described and visually detectable. At the bottom left, we report the accuracy of this model on classifying pre-symptomatic individuals close to HD onset. The actual brain images of two participants are shown.

We tested whether we could correctly classify individuals with pre-symptomatic HD using the anatomic features of individuals with early symptomatic HD. The goal was to use HD as a model to predict conversion to a specific anatomical phenotype rather than to diagnose HD, which can be done precisely by genetic tests. The classifier was built with individuals with early symptoms (*n* = 13) vs. paired controls, and tested in pre-symptomatic individuals close (*n* = 16) and far (*n* = 23) from the onset, vs. paired controls (Supplementary Table). Again, two components were enough to create a model with 73% accuracy in classifying pre-symptomatic individuals near to disease onset (Figure 3). The highest loading weights were in the striatum, as expected, based on the disease physiopathogeny. As described by previous studies, striatum atrophy can barely be determined at the individual level on the pre-symptomatic stage, although it can be detected quantitatively, at the group level, up to 15 years before clinical onset. In addition, the early-symptomatic HD group is anatomically heterogeneous, with some individuals presenting very clear striatum atrophy and others being very close to normal (Figure 3). This indicates that in certain disease types or at certain stages of a disease, the anatomy may not encode enough information to provide diagnosis for all patients. Regardless, we were effective enough in capturing and using this feature for the individual classification. The model did not achieve accuracy significantly higher than the by-chance for classifying pre-symptomatic individuals far from HD onset.

### Alzheimer's disease: classification of diseases with subtle or heterogeneous abnormalities

**Figure 4:**
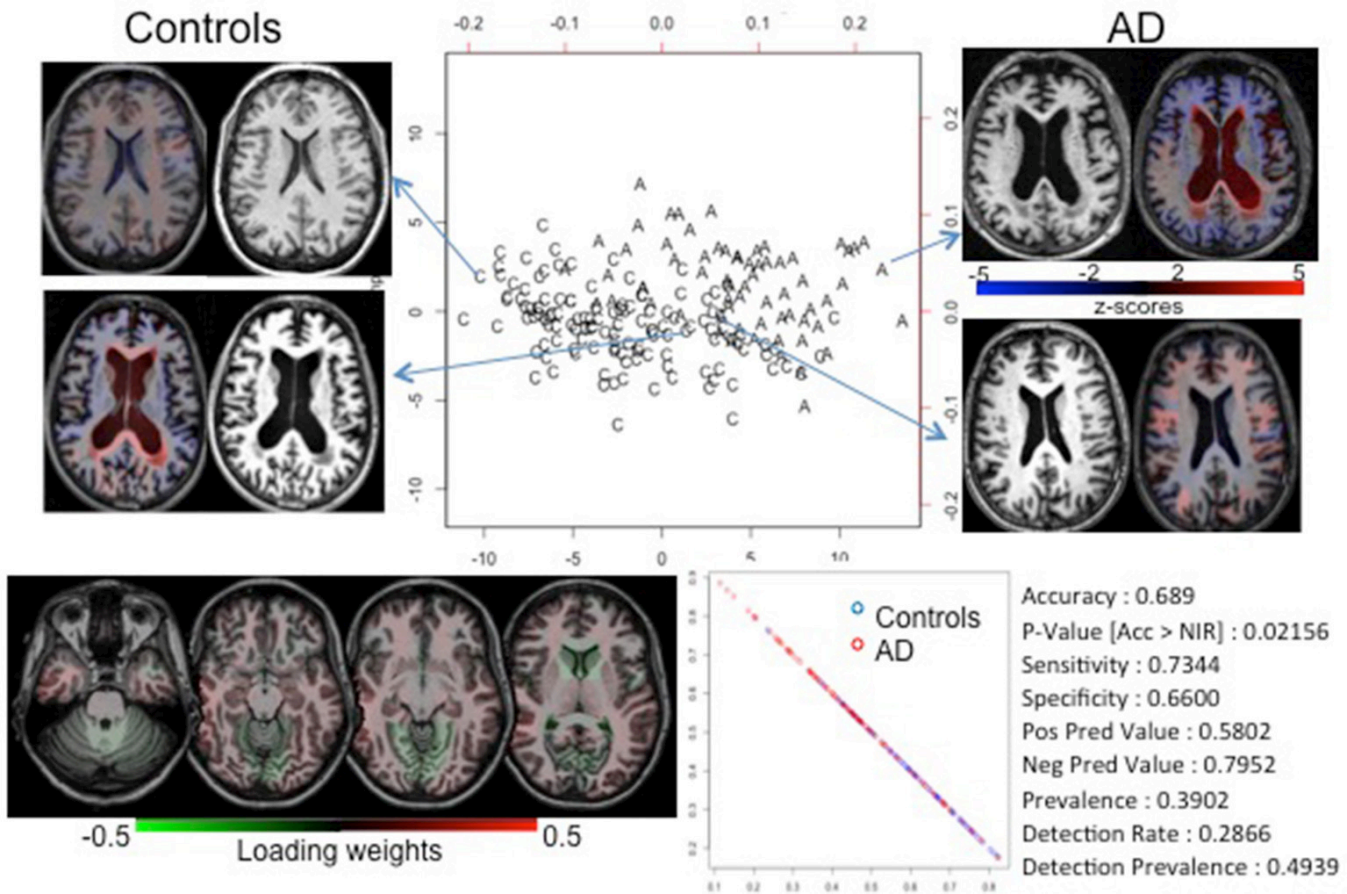
PLS-DA biplot of controls (C) and individuals with AD (A). The overlap between groups is likely due to the heterogeneity and subtleness of imaging features (see the map of loading weights at the bottom left). The anatomical images (brain MRIs) show that individuals at the extremes of the groups show marked anatomical features, while those in the intermediary zone have dubious (both quantitative and qualitative) findings. The colors overlaid in the brain MRIs code the z-scores of the volume (i.e., the regional degree of atrophy); blue is atrophy, red is enlargement. They also show how the quantitative information can be delivered in an understandable way. Using the higher level of granularity, it was possible to create a model with accuracy greater than by chance (bottom right), although lower than in the cases reported before. This was evidenced by the probability plot's showing less segregation between individuals of different groups (bottom center).

Unlike in ataxia and HD, the atrophy in most of the neurodegenerative diseases is detectable at the late stage of the disease and is regionally heterogeneous. This is the case with AD. We achieved a reasonable accuracy (69%) in diagnosing AD (model built in 33 AD individuals vs. paired controls, and tested in independent 33 AD individuals vs. paired controls; see Supplementary Table), significantly higher than the by-chance classification. However, there was an enormous overlap among groups, as notable in the PLS-DA plot and in the probability plot that represents the chance of each individual's belonging to each group (Figure 4). The loading-weights map showed no distinguishing features; the weights are comparable and widespread, indicating that the anatomy in AD is mildly or heterogeneously affected, which can be confirmed by visual inspection of the brain MRIs.

### Primary progressive aphasia: binning by anatomical phenotype

**Figure 5:**
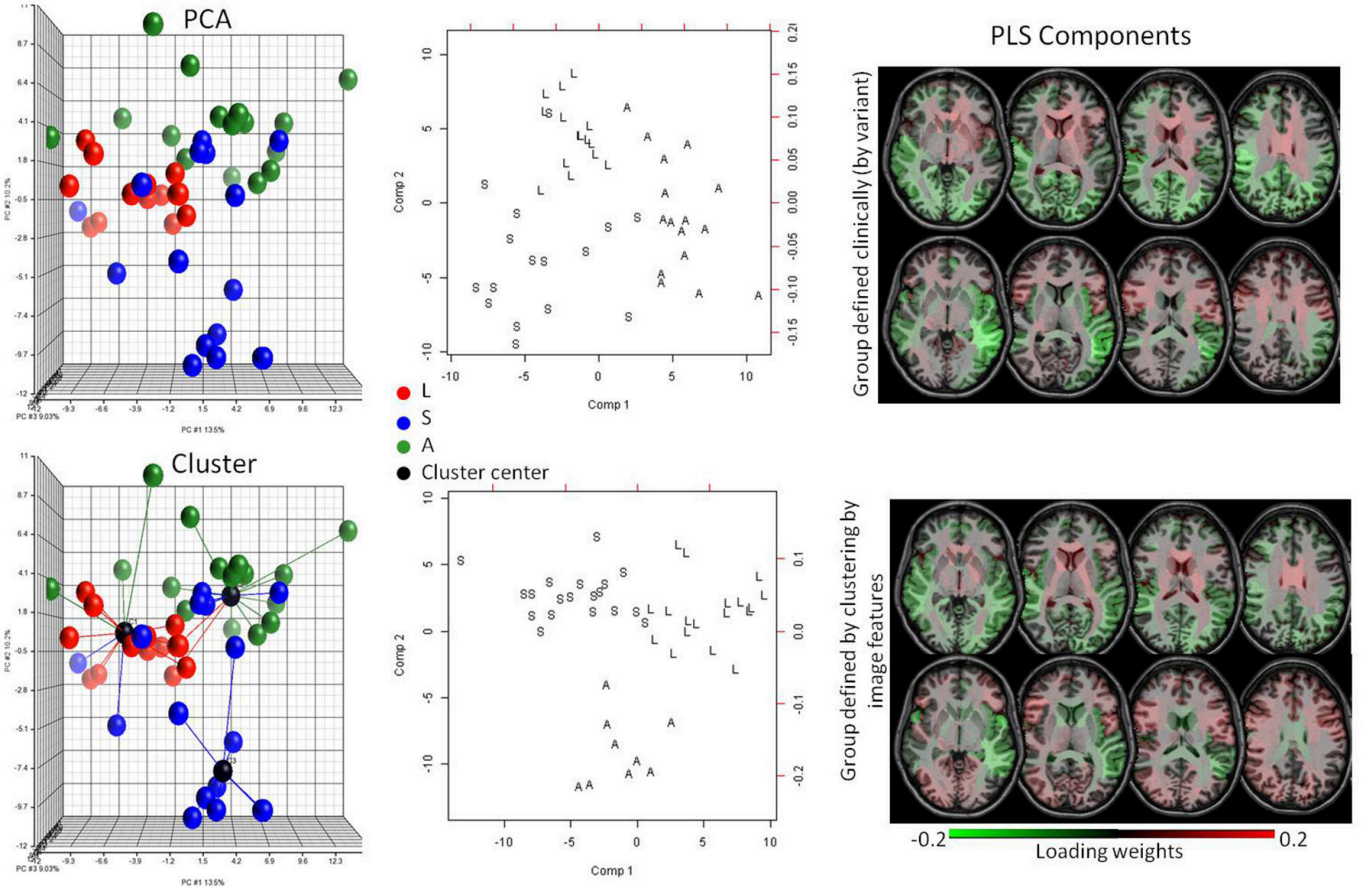
Potential of detecting subgroups in heterogeneous pathologies. The top row is a supervised analysis, with knowledge about the PPA variant; the bottom row is unsupervised, based only on image features. The colors in the plots code three PPA variants (L = logopenic, S = semantic, A = agrammatic). The PCA plot (top left) shows a natural segregation between the variants. Without any clinical information, the images are clustered with high accuracy (Rand Index = 0.71) (bottom left). The anatomical features extracted in the PLS-DA model (center) when patients are grouped by clinical information (top right) or clustered by image features (bottom right) are very similar, and agree with the anatomical features described for the variants, indicating that both methods yield groups based on the same anatomical pattern.

As mentioned in the previous section, increasingly therapies are targeting the early stages of neurodegenerative diseases. However, accurate diagnosis is more difficult because of the lack of clear and/or specific clinical deficits. At this stage, the initial stratification of the heterogeneous patient population is of critical importance. The difficulty arises because potential patient subgroups are degenerate both in the clinical and anatomical domains. In this case, we are interested less in correlation between present diagnosis and anatomical features (because the diagnosis based on clinical information cannot separate important subgroups) and more in expanding the patient populations using both clinical and anatomical manifestations, potentially identifying a way to define subgroups. Binning a disease into subgroups may facilitate the design of therapies and the creation of predictive models because the subgroups may be related to specific pathological substrates, deficits or prognoses. We used PPA as a model system because of the existence of three well-known clinical variants. The knowledge of their anatomical correlates, albeit loose, could serve as our gold standard. In the PCA of the anatomical features (the regional volumes) there was a natural segregation into three clinically labeled groups (Figure 5). By clustering the data using only the anatomical features, we found groups that accurately agreed with the variant diagnosis (Rand Index = 0.71). Then, by using PLS-DA and extracting the loading weights, we confirmed that the features for automated classification according to clusters agreed with those for the classification according to clinical diagnosis. In addition, these anatomical features agreed with what is clinically defined for the variants, such as predominance of atrophy in the left temporal lobe for the Semantic variant, in the inferior parietal for the Logopenic, and in the inferior frontal lobe and the insula for the Agrammatic (Gorno-Tempini et al., 2004).

## Discussion

We evaluated the performance of structure-based computational analysis on extracting anatomical features, previously described by human experience and a priori biological knowledge, in specific patient populations. Previously, we tested the robustness of our automated quantification approach against different image protocols and scanners, using subjects with different patterns and degrees of brain atrophy, and compared our conclusions with those of trained clinicians using visual analysis (Djamanakova et al., 2013; Faria et al., 2015; Liang et al., 2015). In the present study, we tested whether we could classify individuals and anatomically characterize different diseases in simulated clinical scenarios. Our database contains diverse image protocols and scanners. The demographic information taken into account by the linear classifiers include only age and gender, which are always clinically available. Although we could create better classification models by adding other clinical information, homogenizing the dataset, or using classifiers more sophisticated than PLS-DA, this would reduce the potential for generalization and translation to real clinical situations. In summary, rather than the greatness of classification, our aim was to create models robust enough to be translated to clinical practice, and at least in a first step, perform as well as clinicians in terms of extraction of important anatomical features and detection of anatomical patterns, helping to fill the semantic gap.

### Detection of abnormal imaging patterns

In a disease with a clear anatomical phenotype (Ataxia), we obtained 87.5% accuracy, using a small sample size of patients in different stages of the disease. More important, the anatomical features extracted agreed with what is previously described as the hallmark of Ataxia (cerebellar and brainstem atrophy) (Klockgether et al., 1998; Schulz et al., 1999; Eichler et al., 2011; Reetz et al., 2013). The maps of the loading weights and the visual inspection of the images (Figure 2) reveal that the first component carries mostly information about the disease's anatomical phenotype, while the second component basically reflects brain atrophy directly related to age. Thus, the components extracted carry biological meaning, i.e., they contain information that can be interpreted in the light of actual medical knowledge because they both (our quantification tool and the medical knowledge) are based at the level of anatomical structures. In consequence, the classification models and the feature extraction machinery can be easily interpreted and translated to clinical practice. Although this result is purely confirmatory, the quantitative and systematic characterization of the anatomical feature in the PLS-DA space may give us an interesting clue about the patient status. For example, if there are ataxia patients who not only have the typical ataxia feature (component 1), but also are located at an unexpected position in component 2 (i.e., accelerated whole-brain atrophy related to age), this may correlate with poor future outcomes. Thus, a quantitative approach of this type could provide new insight into diagnosis and prognosis, further facilitating research.

To investigate the prognostic value of quantitative anatomical description, we tested the classification performance in diseases where the anatomy clearly correlates with the time course, applying the classifiers in stages where the abnormal features couldn't be detected visually, at the individual level. In other words, we tested the potential for prognostic prediction using the HD population. We achieved 73% accuracy in classifying pre-symptomatic HD individuals, with a model based on features of early symptomatic HD individuals. The feature selection identified the deep gray matter as the most important region for the classification, again agreeing with the physiopathology of HD (Figure 3) (Aylward et al., 2000; Nopoulos et al., 2010; Paulsen et al., 2010, 2014a,b; Guo et al., 2012; Delmaire et al., 2013; Georgiou-Karistianis et al., 2013; Faria et al., 2016). HD is a genetic disease where the product of genetic load and age correlates very well with the time to onset (Ross et al., 2014). Therefore, one can reasonably argue that predictive models based on imaging features are useless. The same applies to Ataxia to some extent. However, our aim was not to diagnosis HD or Ataxia. These diseases were taken as models for proof of concept because the gold standard (clinical diagnosis) is well-established. The aim was to evaluate the structure-based automated quantification approach, in terms of feature selection and robustness against heterogeneous datasets, and its potential to detect features that go beyond the artifactual noise. Particularly in HD, the potential for classifying pre-symptomatic individuals surpasses what can be done with clinical imaging analysis because the subtle abnormalities are not visually detectable at the individual level (Paulsen et al., 2008).

### Potential for binning in more homogeneous phenotypes

Unlike diseases with a clear anatomical phenotype, those that embrace heterogeneous anatomical and clinical phenotypes, or subtle abnormalities, or unknown time courses, offer extra challenges for both visual and automated analysis. This is, for instance, the case with AD. To date, there are about 100 models for predicting conversion from mild cognitive impairment to AD, based on imaging. A PubMed search for “Prediction of MCI to AD conversion MRI” reveals 96 publications in the last 10 years; for more recent reviews, please see (Shaffer et al., 2013; Sanchez-Catasus et al., 2017). Either they achieve unsatisfactory accuracy, or high accuracy at the cost of overfitting, or they are late in the disease course. As a result, we can generalize by saying that there is, as yet, no effective prediction useful in clinical scenarios. Figure 4 may offer some clues about why this happens. Our classification model achieved <70% accuracy. There is substantial overlap between controls and AD in the PLS-DA plot, and there is no predominant weight in the loadings of component 1. Visual inspection of the images reveals that both groups (control and AD) are heterogeneous in terms of atrophy pattern and degree at this age range. This explains why the individual classification, by visual radiological analysis, is also ineffective.

The source of this challenge is two-fold. First, it is possible that anatomy is not encoding enough information to characterize the pathology reliably. Second, because we do not have strongly discriminating factors, both in clinical and imaging information, the stratification of the patient population is incomplete. For example, if AD is actually a syndrome caused by multiple pathologies with multiple anatomical manifestations, AD's common anatomical features cannot be extracted. In this situation, we need to resort to different study designs, using both clinical and imaging features to stratify the population. Models such as AD provide opportunities to investigate the existence of subgroups, with certain anatomical expression, that can behave as specific entities in some clinical domains. For instance, in Ataxia (Figure 2) one can see a subtle spread of patients along the component that differentiates the groups (component 1). Hypothetically, this spread may reflect the effect of a correlated feature, such as disease severity. Similarly in AD or other heterogeneous disease models, there may be a non-orthogonal axis that represents an unknown variable. With regression in this axis, it is possible to detect the subgroups that, for instance, respond differently to therapeutics, or have different prognosis.

To investigate the potential of the automated structure-based quantification to binning an entity into subgroups of clinical relevance, we used individuals with PPA. PPA, a neurodegenerative clinical syndrome characterized by decline in language ability 2 years before any other cognitive deficit, is an ideal condition to investigate the clustering in sub-phenotypes, since three variants loosely correlated with underlying pathologies and with certain anatomical representation are described (Gorno-Tempini et al., 2011; Rohrer and Rosen, 2013). Although there is still no treatment for PPA, there is hope that certain therapies can be effective for specific variants (Cadorio et al., 2017). Now, suppose that the three variants are yet unknown. An unsupervised PCA plot shows a natural segregation of the data into two or three subgroups (Figure 5), but because the variants are hypothetically unknown, one cannot explain the data variance with clinical labels. An unsupervised hierarchical cluster shows the data divided into subgroups that correlate very well with the real variant's diagnosis. The image features selected for classification in these clusters (bottom row, Figure 5) agree with those selected for classification according to the real variant's diagnosis (top row, Figure 5) and also to those that are described as hallmarks for the variants (Turner et al., 1996; Rohrer et al., 2009; Shim et al., 2012; Zhang et al., 2013; Agosta et al., 2015; Botha et al., 2015; Bisenius et al., 2016), proving the potential of our approach to identify subgroups of clinical relevance.

### Deliverables

Subgrouping can be extrapolated to individuals, i.e., the detection of outliers in terms of anatomy may point to individuals who may be unique in additional domains. For instance, in HD (Figure 3) anatomical heterogeneity still remains among the genetically homogenized group, as there is at least one individual with visually normal anatomy. It is an open question if this anatomical variability has any predictive value for prognosis, to be answered by quantitative and systematic characterization of this population.

**Figure 6:**
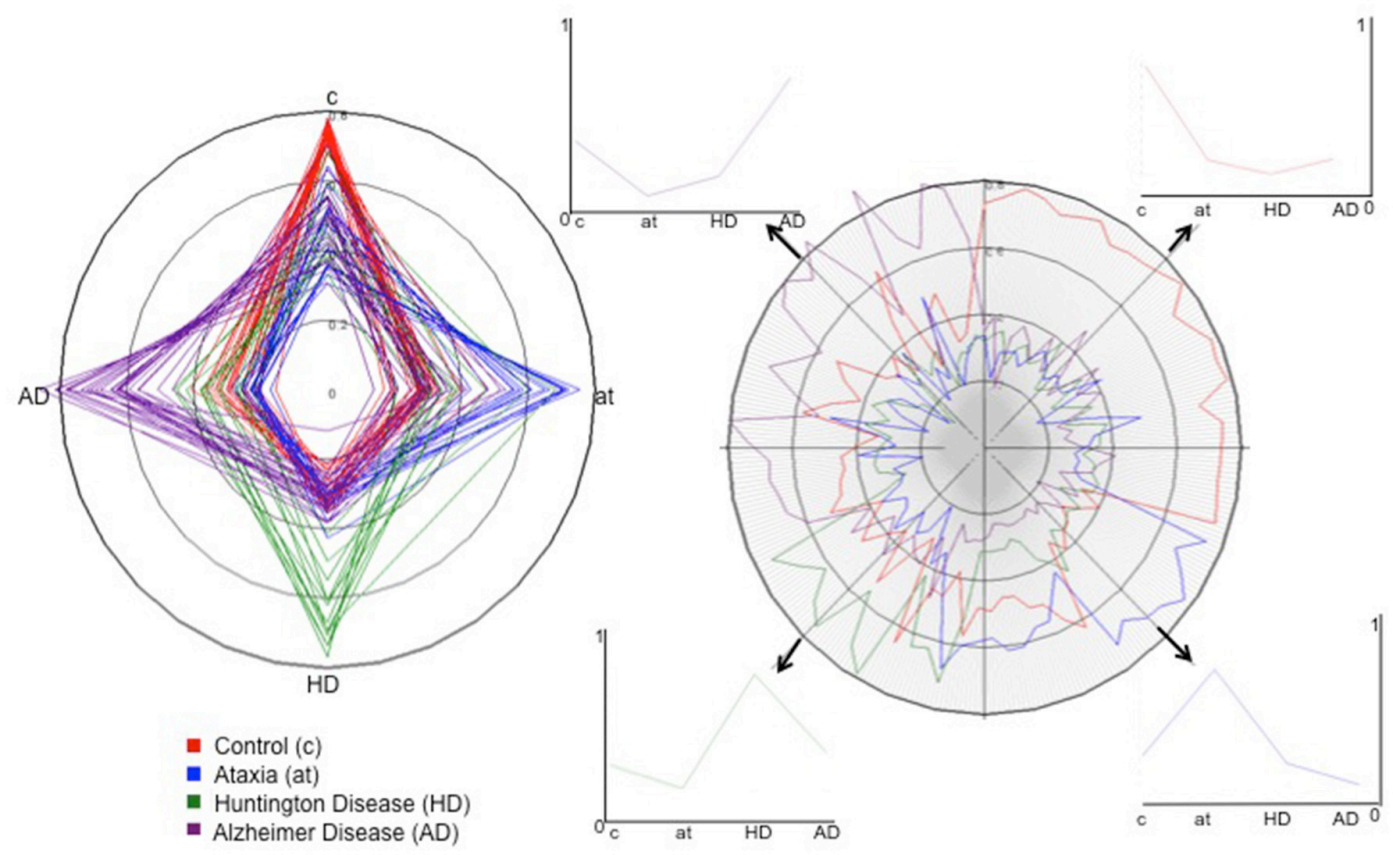
Pattern recognition and probabilistic diagnostic plots. This figure was created by inputting the probability of classification of each individual in different groups, i.e., the individual's chances of belonging to different groups, given by the PLS models. In the star plot (left), each star is an individual, and the colors are their true diagnosis. The x and y axis represent the diagnosis according to our classification models. The point where the stars cross the circles in each axis represents the probability of an individual's being labeled as having the diagnosis coded by that axis. The fact that the stars are elongated where the color (true diagnosis) agrees with the axis diagnosis indicates that the vast majority of patients are correctly classified. At right, a different representation of the same data, easier to visualize the probability of diagnoses in a single individual. Now, the colors represent the diagnosis given by our classification models. Each line represents an individual; the crossing point between the colored lines and the axial lines represents the probability of such an individual's being given that diagnosis. The four small panels at right show the probability curves of diagnosis for four selected individuals (bold arrows), color-coded by the true diagnosis. Y axis ranges from 0 to 1 and encodes the chance of the selected individual of being classified, by the algorithm, with the diagnosis in the X axis. For instance, the individual in the upper left quadrant has almost no chance (close to 0) of being classified as AT, a low chance of being classified as HD, a higher chance of being classified as control, and a high chance of being classified as AD. In fact, this individual had AD, as revealed by the color (purple) that represents the true diagnosis.

Another potential deliverable is the diagnostic probability map for each individual (Figure 6). Given a database large enough to contain various pathologies and the high variability of imaging protocols and age range for controls and patients, it is possible to calculate the probability of differential diagnosis for a new individual, as shown in Figure 6. In this example, one can reasonably argue that it is clinically improbable to have HD, Ataxia and AD as differential diagnoses. Again, these diseases were taken as proof of concept, because they all have the same basic anatomic feature (atrophy) and a clear clinical diagnosis used as the gold-standard. The concept of diagnostic probability graphics can be extended to more plausible clinical scenarios.

Finally, the potential for aiding clinical interpretation and education may be a valuable low-hanging fruit. The simple use of z-score maps (Figure 4) may confirm or exclude a clinical impression and speed up the radiological reading. Also, having a database big and heterogeneous enough, and coupling image and text information (such as diagnosis, prognosis, response to treatments, etc.), it is possible to perform a direct image search, producing static reports about similar phenotypes. For example, given a new subject image, it is possible to search in a big dataset for dozens of images with similar features linked to information of clinical relevance.

### Limitations

This study is based on a single image variable, the volume. One of the greatest advantages of the structure-based approach is that it allows the combination of many other features, such as T2 contrast, diffusion tensor image indices, functional MRI correlations, metabolite concentration, and others, as we demonstrated in previous studies (Faria et al., 2012). Although there are big challenges in combining features of different domains (e.g., drawbacks on feature concatenation methods, variation among clinical protocols barring the creation of common databases for certain domains, the need for a priori knowledge of noise in order to create models for easy generalization), multi-domain structure-based analysis is a promising strategy for conditions with no single dominant discriminating feature.

An important constraint of the structure-based analysis is that any quantitative characterization and classification model will be limited by the pre-defined space. In other words, if the anatomical pattern does not respect the boundaries of a given parcellation scheme, the abnormality can be overlooked. One strategy to ameliorate this issue is to use different levels of granularity. So one can analyze the data in parcels as big as a hemisphere, or as small as a cranial nerve, which is actually smaller than the gaussian filters traditionally used for voxel-based analysis. However, if the strategy is to replicate the radiological interpretation, then structure-based analysis is intuitively a better solution because visual inspection occurs at the structural level, not at the voxel level.

### Perspectives

We explored the performance of structure-based computational analysis in simulated clinical scenarios. The pillars of this approach are automated and accurate quantification, reliability and robustness against artifactual noise, easy interpretation of selected features, and a knowledge repository that is a large database as heterogeneous as possible both in terms of pathologies and image protocols. The deliverables are diverse, from the image quantification itself, through to the image pattern search, to the diagnostic aid. Although the impact of this method is yet to be tested, it has potential educational value, it may reduce the time for radiological reading, or it may work as second reader in locations where sub-specialized radiologists are not available. In any case, because no such tool can be directly applicable to clinical practice, any positive impact is valuable. In addition, electronic structurized databases and search engines are the basis of high throughput image analysis and may represent the migration of brain MRI to the BigData era, contributing to the emergent field of Precision Medicine.

## Ethics statement

This study was carried out with data (1) from publically available databases, and (2) from Johns Hopkins Hospital, originally collected for other studies. These studies were approved and carried out in accordance with the recommendations of the local IRB, with written informed consent from all subjects, in accordance with the Declaration of Helsinki.

## Author contributions

All the authors are accountable for all aspects of the work and approved the final version. In addition, AF and SM are responsible for the conception and design, interpretation of data, and drafting the work. ZL is responsible for data acquisition and analysis. MIM revised it critically for important intellectual content.

### Conflict of interest statement

SM and MM own AnatomyWorks. SM is its CEO. This arrangement is being managed by the Johns Hopkins University in accordance with its conflict-of-interest policies. The other authors declare that the research was conducted in the absence of any commercial or financial relationships that could be construed as a potential conflict of interest.
