# Supplementary material for: Brain MRI Pattern Recognition Translated to Clinical Scenarios
Source: Front Neurosci. 2017 Oct 20;11:578. doi: 10.3389/fnins.2017.00578 (PMC5655969; doi:10.3389/fnins.2017.00578)
Supplement: Supplementary file 1 [file DataSheet1.docx]

**Supplementary Table:** Demographics and covariate statistics for the groups in used in the PLS analysis

| Statistical Summary | controls age (sd) | "disease" age (sd) | p-value (ttest-age) | controls male/ female | "Disease" male/ female | p-value (Fisher gender) | p-value (Fisher protocol) |
| --- | --- | --- | --- | --- | --- | --- | --- |
| HD early symptoms vs. controls | 45.1±6.5 | 50.8±7.9 | 0.05 | 8/5 | 7/6 | 1 | 1 |
| HD near to onset vs. controls | 38.6±9.2 | 45.1±8.6 | 0.05 | 10/6 | 13/3 | 0.43 | 1 |
| HD far to onset vs. controls | 38.2±9.2 | 36.8±9.7 | 0.67 | 11/6 | 10/13 | 0.74 | 1 |
| Ataxia vs. controls | 57.4±3.7 | 60.8±6.8 | 0.09 | 10/5 | 13/3 | 0.41 | 1 |
| AD vs. controls | 76.2±9.2 | 74.1±10.5 | 0.24 | 38/28 | 40/26 | 0.85 | 0.35 |

| group | gender | age | protocol |
| --- | --- | --- | --- |
| HD_control | M | 50 | Phillips, 3T, 0.9x0.9x0.9 |
| HD_control | M | 47 | Phillips, 3T, 0.9x0.9x0.9 |
| HD_control | M | 53 | Phillips, 3T, 0.9x0.9x0.9 |
| HD_control | M | 43 | Phillips, 3T, 0.9x0.9x0.9 |
| HD_control | F | 37 | Phillips, 3T, 0.9x0.9x0.9 |
| HD_control | M | 33 | Phillips, 3T, 0.9x0.9x0.9 |
| HD_control | F | 49 | Phillips, 3T, 0.9x0.9x0.9 |
| HD_control | F | 50 | Phillips, 3T, 0.9x0.9x0.9 |
| HD_control | F | 48 | Phillips, 3T, 0.9x0.9x0.9 |
| HD_control | F | 48 | Phillips, 3T, 0.9x0.9x0.9 |
| HD_control | M | 45 | Phillips, 3T, 0.9x0.9x0.9 |
| HD_control | M | 49 | Phillips, 3T, 0.9x0.9x0.9 |
| HD_control | M | 34 | Phillips, 3T, 0.9x0.9x0.9 |
| HD_EarlySymptoms | M | 54 | Phillips, 3T, 0.9x0.9x0.9 |
| HD_EarlySymptoms | M | 59 | Phillips, 3T, 0.9x0.9x0.9 |
| HD_EarlySymptoms | F | 42 | Phillips, 3T, 0.9x0.9x0.9 |
| HD_EarlySymptoms | F | 56 | Phillips, 3T, 0.9x0.9x0.9 |
| HD_EarlySymptoms | M | 49 | Phillips, 3T, 0.9x0.9x0.9 |
| HD_EarlySymptoms | M | 47 | Phillips, 3T, 0.9x0.9x0.9 |
| HD_EarlySymptoms | M | 58 | Phillips, 3T, 0.9x0.9x0.9 |
| HD_EarlySymptoms | F | 53 | Phillips, 3T, 0.9x0.9x0.9 |
| HD_EarlySymptoms | F | 49 | Phillips, 3T, 0.9x0.9x0.9 |
| HD_EarlySymptoms | F | 57 | Phillips, 3T, 0.9x0.9x0.9 |
| HD_EarlySymptoms | M | 30 | Phillips, 3T, 0.9x0.9x0.9 |
| HD_EarlySymptoms | F | 52 | Phillips, 3T, 0.9x0.9x0.9 |
| HD_EarlySymptoms | M | 55 | Phillips, 3T, 0.9x0.9x0.9 |

| group | gender | age | protocol |
| --- | --- | --- | --- |
| HD_control | M | 49 | Phillips, 3T, 0.9x0.9x0.9 |
| HD_control | M | 49 | Phillips, 3T, 0.9x0.9x0.9 |
| HD_control | F | 28 | Phillips, 3T, 0.9x0.9x0.9 |
| HD_control | M | 31 | Phillips, 3T, 0.9x0.9x0.9 |
| HD_control | M | 53 | Phillips, 3T, 0.9x0.9x0.9 |
| HD_control | M | 28 | Phillips, 3T, 0.9x0.9x0.9 |
| HD_control | M | 30 | Phillips, 3T, 0.9x0.9x0.9 |
| HD_control | F | 37 | Phillips, 3T, 0.9x0.9x0.9 |
| HD_control | M | 33 | Phillips, 3T, 0.9x0.9x0.9 |
| HD_control | F | 49 | Phillips, 3T, 0.9x0.9x0.9 |
| HD_control | F | 50 | Phillips, 3T, 0.9x0.9x0.9 |
| HD_control | F | 28 | Phillips, 3T, 0.9x0.9x0.9 |
| HD_control | F | 40 | Phillips, 3T, 0.9x0.9x0.9 |
| HD_control | M | 45 | Phillips, 3T, 0.9x0.9x0.9 |
| HD_control | M | 33 | Phillips, 3T, 0.9x0.9x0.9 |
| HD_control | M | 35 | Phillips, 3T, 0.9x0.9x0.9 |
| HD_neartoOneset | M | 39 | Phillips, 3T, 0.9x0.9x0.9 |
| HD_neartoOneset | F | 52 | Phillips, 3T, 0.9x0.9x0.9 |
| HD_neartoOneset | M | 52 | Phillips, 3T, 0.9x0.9x0.9 |
| HD_neartoOneset | M | 47 | Phillips, 3T, 0.9x0.9x0.9 |
| HD_neartoOneset | M | 38 | Phillips, 3T, 0.9x0.9x0.9 |
| HD_neartoOneset | F | 44 | Phillips, 3T, 0.9x0.9x0.9 |
| HD_neartoOneset | F | 51 | Phillips, 3T, 0.9x0.9x0.9 |
| HD_neartoOneset | M | 53 | Phillips, 3T, 0.9x0.9x0.9 |
| HD_neartoOneset | M | 42 | Phillips, 3T, 0.9x0.9x0.9 |
| HD_neartoOneset | M | 45 | Phillips, 3T, 0.9x0.9x0.9 |
| HD_neartoOneset | M | 51 | Phillips, 3T, 0.9x0.9x0.9 |
| HD_neartoOneset | M | 44 | Phillips, 3T, 0.9x0.9x0.9 |
| HD_neartoOneset | M | 19 | Phillips, 3T, 0.9x0.9x0.9 |
| HD_neartoOneset | M | 43 | Phillips, 3T, 0.9x0.9x0.9 |
| HD_neartoOneset | M | 55 | Phillips, 3T, 0.9x0.9x0.9 |
| HD_neartoOneset | M | 47 | Phillips, 3T, 0.9x0.9x0.9 |

| group | gender | age | protocol |
| --- | --- | --- | --- |
| HD_control | M | 49 | Phillips, 3T, 0.9x0.9x0.9 |
| HD_control | M | 49 | Phillips, 3T, 0.9x0.9x0.9 |
| HD_control | F | 28 | Phillips, 3T, 0.9x0.9x0.9 |
| HD_control | M | 31 | Phillips, 3T, 0.9x0.9x0.9 |
| HD_control | M | 30 | Phillips, 3T, 0.9x0.9x0.9 |
| HD_control | M | 53 | Phillips, 3T, 0.9x0.9x0.9 |
| HD_control | M | 28 | Phillips, 3T, 0.9x0.9x0.9 |
| HD_control | M | 30 | Phillips, 3T, 0.9x0.9x0.9 |
| HD_control | F | 37 | Phillips, 3T, 0.9x0.9x0.9 |
| HD_control | M | 33 | Phillips, 3T, 0.9x0.9x0.9 |
| HD_control | F | 49 | Phillips, 3T, 0.9x0.9x0.9 |
| HD_control | F | 50 | Phillips, 3T, 0.9x0.9x0.9 |
| HD_control | F | 28 | Phillips, 3T, 0.9x0.9x0.9 |
| HD_control | F | 40 | Phillips, 3T, 0.9x0.9x0.9 |
| HD_control | M | 45 | Phillips, 3T, 0.9x0.9x0.9 |
| HD_control | M | 33 | Phillips, 3T, 0.9x0.9x0.9 |
| HD_control | M | 35 | Phillips, 3T, 0.9x0.9x0.9 |
| HD_farfromOneset | M | 26 | Phillips, 3T, 0.9x0.9x0.9 |
| HD_farfromOneset | M | 39 | Phillips, 3T, 0.9x0.9x0.9 |
| HD_farfromOneset | F | 51 | Phillips, 3T, 0.9x0.9x0.9 |
| HD_farfromOneset | F | 30 | Phillips, 3T, 0.9x0.9x0.9 |
| HD_farfromOneset | F | 28 | Phillips, 3T, 0.9x0.9x0.9 |
| HD_farfromOneset | F | 42 | Phillips, 3T, 0.9x0.9x0.9 |
| HD_farfromOneset | M | 33 | Phillips, 3T, 0.9x0.9x0.9 |
| HD_farfromOneset | F | 43 | Phillips, 3T, 0.9x0.9x0.9 |
| HD_farfromOneset | F | 48 | Phillips, 3T, 0.9x0.9x0.9 |
| HD_farfromOneset | F | 50 | Phillips, 3T, 0.9x0.9x0.9 |
| HD_farfromOneset | F | 21 | Phillips, 3T, 0.9x0.9x0.9 |
| HD_farfromOneset | F | 31 | Phillips, 3T, 0.9x0.9x0.9 |
| HD_farfromOneset | F | 49 | Phillips, 3T, 0.9x0.9x0.9 |
| HD_farfromOneset | M | 50 | Phillips, 3T, 0.9x0.9x0.9 |
| HD_farfromOneset | F | 30 | Phillips, 3T, 0.9x0.9x0.9 |
| HD_farfromOneset | F | 29 | Phillips, 3T, 0.9x0.9x0.9 |
| HD_farfromOneset | M | 35 | Phillips, 3T, 0.9x0.9x0.9 |
| HD_farfromOneset | M | 43 | Phillips, 3T, 0.9x0.9x0.9 |
| HD_farfromOneset | M | 22 | Phillips, 3T, 0.9x0.9x0.9 |
| HD_farfromOneset | M | 31 | Phillips, 3T, 0.9x0.9x0.9 |
| HD_farfromOneset | F | 37 | Phillips, 3T, 0.9x0.9x0.9 |
| HD_farfromOneset | M | 50 | Phillips, 3T, 0.9x0.9x0.9 |
| HD_farfromOneset | M | 29 | Phillips, 3T, 0.9x0.9x0.9 |

| group | gender | age | protocol |
| --- | --- | --- | --- |
| AT_control | M | 62 | Phillips, 3T, 1.1x0.83x0.83x0.83 |
| AT_control | F | 47 | Phillips, 3T, 1.1x0.83x0.83x0.83 |
| AT_control | M | 59 | Phillips, 3T, 1.1x0.83x0.83x0.83 |
| AT_control | F | 58 | Phillips, 3T, 1.1x0.83x0.83x0.83 |
| AT_control | F | 54 | Phillips, 3T, 1.1x0.83x0.83x0.83 |
| AT_control | M | 60 | Phillips, 3T, 1.1x0.83x0.83x0.83 |
| AT_control | M | 59 | Phillips, 3T, 1.1x0.83x0.83x0.83 |
| AT_control | M | 58 | Phillips, 3T, 1.1x0.83x0.83x0.83 |
| AT_control | F | 57 | Phillips, 3T, 1.1x0.83x0.83x0.83 |
| AT_control | M | 59 | Phillips, 3T, 1.1x0.83x0.83x0.83 |
| AT_control | M | 58 | Phillips, 3T, 1.1x0.83x0.83x0.83 |
| AT_control | F | 55 | Phillips, 3T, 1.1x0.83x0.83x0.83 |
| AT_control | M | 62 | Phillips, 3T, 1.1x0.83x0.83x0.83 |
| AT_control | M | 55 | Phillips, 3T, 1.1x0.83x0.83x0.83 |
| AT_control | M | 58 | Phillips, 3T, 1.1x0.83x0.83x0.83 |
| AT | M | 73 | Phillips, 3T, 1.1x0.83x0.83x0.83 |
| AT | M | 48 | Phillips, 3T, 1.1x0.83x0.83x0.83 |
| AT | M | 53 | Phillips, 3T, 1.1x0.83x0.83x0.83 |
| AT | M | 62 | Phillips, 3T, 1.1x0.83x0.83x0.83 |
| AT | M | 56 | Phillips, 3T, 1.1x0.83x0.83x0.83 |
| AT | M | 56 | Phillips, 3T, 1.1x0.83x0.83x0.83 |
| AT | M | 53 | Phillips, 3T, 1.1x0.83x0.83x0.83 |
| AT | M | 56 | Phillips, 3T, 1.1x0.83x0.83x0.83 |
| AT | F | 63 | Phillips, 3T, 1.1x0.83x0.83x0.83 |
| AT | M | 63 | Phillips, 3T, 1.1x0.83x0.83x0.83 |
| AT | F | 67 | Phillips, 3T, 1.1x0.83x0.83x0.83 |
| AT | M | 67 | Phillips, 3T, 1.1x0.83x0.83x0.83 |
| AT | F | 68 | Phillips, 3T, 1.1x0.83x0.83x0.83 |
| AT | M | 68 | Phillips, 3T, 1.1x0.83x0.83x0.83 |
| AT | M | 60 | Phillips, 3T, 1.1x0.83x0.83x0.83 |
| AT | M | 60 | Phillips, 3T, 1.1x0.83x0.83x0.83 |

| group | gender | age | protocol |
| --- | --- | --- | --- |
| AD_Controls | F | 70 | SIEMENS, 3T, 1.2x1x1 |
| AD_Controls | F | 71 | SIEMENS, 3T, 1.2x1x1 |
| AD_Controls | F | 55 | SIEMENS, 3T, 1.2x1x1 |
| AD_Controls | F | 70 | SIEMENS, 3T, 1.2x1x1 |
| AD_Controls | M | 78 | SIEMENS, 3T, 1.2x1x1 |
| AD_Controls | F | 72 | SIEMENS, 3T, 1.2x1x1 |
| AD_Controls | F | 76 | SIEMENS, 3T, 1.2x1x1 |
| AD_Controls | M | 80 | SIEMENS, 3T, 1.2x1x1 |
| AD_Controls | M | 75 | SIEMENS, 3T, 1.2x1x1 |
| AD_Controls | F | 77 | SIEMENS, 3T, 1.2x1x1 |
| AD_Controls | M | 75 | SIEMENS, 3T, 1.2x1x1 |
| AD_Controls | M | 80 | SIEMENS, 3T, 1.2x1x1 |
| AD_Controls | F | 80 | SIEMENS, 3T, 1.2x1x1 |
| AD_Controls | M | 78 | SIEMENS, 3T, 1.2x1x1 |
| AD_Controls | M | 85 | SIEMENS, 3T, 1.2x1x1 |
| AD_Controls | F | 79 | SIEMENS, 3T, 1.2x1x1 |
| AD_Controls | M | 67 | SIEMENS, 3T, 1.2x1x1 |
| AD_Controls | M | 71 | SIEMENS, 3T, 1.2x1x1 |
| AD_Controls | M | 75.5 | SIEMENS, 3T, 1.2x1x1 |
| AD_Controls | M | 85 | SIEMENS, 3T, 1.2x1x1 |
| AD_Controls | F | 75.7 | SIEMENS, 3T, 1.2x1x1 |
| AD_Controls | F | 85.3 | SIEMENS, 3T, 1.2x1x1 |
| AD_Controls | M | 65.2 | SIEMENS, 3T, 1.2x1x1 |
| AD_Controls | F | 95.3 | SIEMENS, 1.5T, 1.2x1.25x1.25 |
| AD_Controls | M | 90.4 | SIEMENS, 1.5T, 1.2x1.25x1.25 |
| AD_Controls | M | 83.3 | SIEMENS, 1.5T, 1.2x1.25x1.25 |
| AD_Controls | M | 65.1 | SIEMENS, 1.5T, 1.2x1.25x1.25 |
| AD_Controls | F | 65.3 | SIEMENS, 1.5T, 1.2x1.25x1.25 |
| AD_Controls | F | 75.9 | SIEMENS, 1.5T, 1.2x1.25x1.25 |
| AD_Controls | M | 77 | SIEMENS, 1.5T, 1.2x1.25x1.25 |
| AD_Controls | M | 74 | Phillips, 3T, 1.2x1x1 |
| AD_Controls | M | 70 | Phillips, 3T, 1.2x1x1 |
| AD_Controls | M | 91 | Phillips, 3T, 1.2x1x1 |
| AD_Controls | F | 83.4 | Phillips, 3T, 1.2x1x1 |
| AD_Controls | F | 94.7 | Phillips, 3T, 1.2x1x1 |
| AD_Controls | F | 76.3 | Phillips, 3T, 1.2x1x1 |
| AD_Controls | M | 75.1 | Phillips, 3T, 1.2x1x1 |
| AD_Controls | F | 58.5 | Phillips, 3T, 1.2x1x1 |
| AD_Controls | M | 85 | Phillips, 3T, 1.2x1x1 |
| AD_Controls | F | 65.2 | Phillips, 3T, 1.2x1x1 |
| AD_Controls | M | 70 | Phillips, 3T, 1.2x1x1 |
| AD_Controls | M | 66 | Phillips, 3T, 1.2x1x1 |
| AD_Controls | M | 81 | Phillips, 3T, 1.2x1x1 |
| AD_Controls | M | 75 | Phillips, 3T, 1.2x1x1 |
| AD_Controls | M | 67 | Phillips, 3T, 1.2x1x1 |
| AD_Controls | M | 60 | Phillips, 3T, 1.2x1x1 |
| AD_Controls | F | 56.3 | Phillips, 3T, 1.2x1x1 |
| AD_Controls | M | 66.2 | Phillips, 1.5T, 1.2x0.94x0.94 |
| AD_Controls | M | 75.9 | Phillips, 1.5T, 1.2x0.94x0.94 |
| AD_Controls | F | 80.6 | Phillips, 1.5T, 1.2x0.94x0.94 |
| AD_Controls | F | 75.4 | Phillips, 1.5T, 1.2x0.94x0.94 |
| AD_Controls | M | 80.6 | Phillips, 1.5T, 1.2x0.94x0.94 |
| AD_Controls | M | 90 | Phillips, 1.5T, 1.2x0.94x0.94 |
| AD_Controls | M | 68 | Phillips, 1.5T, 1.2x0.94x0.94 |
| AD_Controls | F | 88 | GE, 3T, 1.2x1.02x1.02 |
| AD_Controls | M | 85.1 | GE, 3T, 1.2x1.02x1.02 |
| AD_Controls | M | 64.8 | GE, 3T, 1.2x1.02x1.02 |
| AD_Controls | F | 69.4 | GE, 3T, 1.2x1.02x1.02 |
| AD_Controls | F | 74.7 | GE, 3T, 1.2x1.02x1.02 |
| AD_Controls | M | 75.1 | GE, 3T, 1.2x1.02x1.02 |
| AD_Controls | F | 93.8 | GE, 1.5T, 1.2x0.94x0.94 |
| AD_Controls | M | 84.6 | GE, 1.5T, 1.2x0.94x0.94 |
| AD_Controls | M | 76 | GE, 1.5T, 1.2x0.94x0.94 |
| AD_Controls | M | 90.6 | GE, 1.5T, 1.2x0.94x0.94 |
| AD_Controls | F | 75.7 | GE, 1.5T, 1.2x0.94x0.94 |
| AD_Controls | F | 85.7 | GE, 1.5T, 1.2x0.94x0.94 |
| AD | M | 86 | SIEMENS, 3T, 1.2x1x1 |
| AD | M | 81 | SIEMENS, 3T, 1.2x1x1 |
| AD | F | 77 | SIEMENS, 3T, 1.2x1x1 |
| AD | M | 82 | SIEMENS, 3T, 1.2x1x1 |
| AD | M | 71 | SIEMENS, 3T, 1.2x1x1 |
| AD | M | 82 | SIEMENS, 3T, 1.2x1x1 |
| AD | F | 69 | SIEMENS, 3T, 1.2x1x1 |
| AD | M | 77 | SIEMENS, 3T, 1.2x1x1 |
| AD | M | 75 | SIEMENS, 3T, 1.2x1x1 |
| AD | M | 80 | SIEMENS, 3T, 1.2x1x1 |
| AD | M | 80 | SIEMENS, 3T, 1.2x1x1 |
| AD | F | 56 | SIEMENS, 3T, 1.2x1x1 |
| AD | M | 78 | SIEMENS, 3T, 1.2x1x1 |
| AD | M | 70 | SIEMENS, 3T, 1.2x1x1 |
| AD | M | 78 | SIEMENS, 3T, 1.2x1x1 |
| AD | F | 69 | SIEMENS, 3T, 1.2x1x1 |
| AD | M | 68 | SIEMENS, 3T, 1.2x1x1 |
| AD | M | 76 | SIEMENS, 3T, 1.2x1x1 |
| AD | F | 75.6 | SIEMENS, 3T, 1.2x1x1 |
| AD | M | 56.6 | SIEMENS, 3T, 1.2x1x1 |
| AD | M | 75.8 | SIEMENS, 3T, 1.2x1x1 |
| AD | M | 58.2 | SIEMENS, 3T, 1.2x1x1 |
| AD | M | 85 | SIEMENS, 3T, 1.2x1x1 |
| AD | M | 64.7 | SIEMENS, 3T, 1.2x1x1 |
| AD | F | 85.7 | SIEMENS, 3T, 1.2x1x1 |
| AD | M | 64.9 | SIEMENS, 3T, 1.2x1x1 |
| AD | F | 93 | SIEMENS, 3T, 1.2x1x1 |
| AD | M | 85.2 | SIEMENS, 1.5T, 1.2x1.25x1.25 |
| AD | M | 85.1 | SIEMENS, 1.5T, 1.2x1.25x1.25 |
| AD | M | 75 | SIEMENS, 1.5T, 1.2x1.25x1.25 |
| AD | F | 93 | SIEMENS, 1.5T, 1.2x1.25x1.25 |
| AD | M | 64.9 | SIEMENS, 1.5T, 1.2x1.25x1.25 |
| AD | F | 64.8 | SIEMENS, 1.5T, 1.2x1.25x1.25 |
| AD | F | 56.6 | SIEMENS, 1.5T, 1.2x1.25x1.25 |
| AD | M | 84.4 | Phillips, 3T, 1.2x1x1 |
| AD | F | 75.1 | Phillips, 3T, 1.2x1x1 |
| AD | M | 66.6 | Phillips, 3T, 1.2x1x1 |
| AD | F | 74.9 | Phillips, 3T, 1.2x1x1 |
| AD | F | 84.2 | Phillips, 3T, 1.2x1x1 |
| AD | M | 64 | Phillips, 3T, 1.2x1x1 |
| AD | F | 56 | Phillips, 3T, 1.2x1x1 |
| AD | M | 57 | Phillips, 3T, 1.2x1x1 |
| AD | M | 65 | Phillips, 1.5T, 1.2x0.94x0.94 |
| AD | M | 64.7 | Phillips, 1.5T, 1.2x0.94x0.94 |
| AD | M | 84.9 | Phillips, 1.5T, 1.2x0.94x0.94 |
| AD | F | 84 | Phillips, 1.5T, 1.2x0.94x0.94 |
| AD | M | 59.8 | Phillips, 1.5T, 1.2x0.94x0.94 |
| AD | F | 74.3 | Phillips, 1.5T, 1.2x0.94x0.94 |
| AD | F | 71.5 | Phillips, 1.5T, 1.2x0.94x0.94 |
| AD | F | 82.7 | GE, 3T, 1.2x1.02x1.02 |
| AD | M | 77.1 | GE, 3T, 1.2x1.02x1.02 |
| AD | M | 84.3 | GE, 3T, 1.2x1.02x1.02 |
| AD | F | 77.6 | GE, 3T, 1.2x1.02x1.02 |
| AD | F | 92.4 | GE, 3T, 1.2x1.02x1.02 |
| AD | F | 90.4 | GE, 3T, 1.2x1.02x1.02 |
| AD | M | 64.9 | GE, 3T, 1.2x1.02x1.02 |
| AD | M | 66 | GE, 3T, 1.2x1.02x1.02 |
| AD | F | 74.9 | GE, 1.5T, 1.2x0.94x0.94 |
| AD | M | 84.6 | GE, 1.5T, 1.2x0.94x0.94 |
| AD | M | 64.6 | GE, 1.5T, 1.2x0.94x0.94 |
| AD | F | 55.2 | GE, 1.5T, 1.2x0.94x0.94 |
| AD | F | 84.7 | GE, 1.5T, 1.2x0.94x0.94 |
| AD | M | 75.8 | GE, 1.5T, 1.2x0.94x0.94 |
| AD | M | 65 | GE, 1.5T, 1.2x0.94x0.94 |
| AD | F | 91.3 | GE, 1.5T, 1.2x0.94x0.94 |
| AD | F | 56.5 | GE, 1.5T, 1.2x0.94x0.94 |
